# Bioequivalence and the food effect of macitentan/tadalafil 10/20 fixed‐dose combination tablets versus the use of single‐component tablets in healthy subjects

**DOI:** 10.1002/prp2.1202

**Published:** 2024-05-19

**Authors:** Jennifer Lynn Ford, Ahad Sabet, Jaya Natarajan, Hans Stieltjes, Daniel L. Chao, Navin Goyal, Denes Csonka

**Affiliations:** ^1^ Janssen Research & Development Spring House Pennsylvania USA; ^2^ ICON Salt Lake City Utah USA; ^3^ Janssen Research & Development Raritan New Jersey USA; ^4^ Janssen Research & Development Beerse Belgium; ^5^ Actelion Pharmaceuticals, A Janssen Pharmaceutical Company of Johnson & Johnson Allschwil Switzerland

**Keywords:** bioequivalence, fixed‐dose combination, food effect, macitentan, pulmonary arterial hypertension, tadalafil

## Abstract

The primary aim was to demonstrate bioequivalence between the 10/20 mg fixed‐dose combination (FDC) of macitentan/tadalafil in a single tablet and the free combination of both drugs, and to evaluate the food effect on the 10/20 mg FDC in healthy participants. In this single‐center, randomized, open‐label, 3‐way crossover, single‐dose Phase 1 study in healthy adult participants, macitentan/tadalafil was administered as a 10/20 mg FDC formulation and compared with the free combination of macitentan and tadalafil. The food effect on the FDC was also evaluated. Pharmacokinetic sampling (216 h) was conducted. The 90% confidence intervals (CIs) for the geometric mean ratios of maximum observed plasma analyte concentration (*C*_max_) and area under the plasma analyte concentration–time curves (AUCs) for Treatment A (FDC, fasted) versus C (free combination, fasted) were within bioequivalence limits demonstrating that the FDC formulation can be considered bioequivalent to the free combination. The 90% CIs for the geometric mean ratios of *C*_max_ and AUC for Treatment B (FDC, fed) versus A (FDC, fasted) were contained within bioequivalence limits demonstrating that there was no food effect. The administration of the 10/20 mg FDC was generally safe and well tolerated in healthy participants. This study demonstrated bioequivalence between the FDC of macitentan/tadalafil (10/20 mg) in a single tablet and the free combination of both drugs in healthy participants, and that the FDC can be taken without regard to food, similarly to the individual components. The FDC was generally safe and well tolerated.

## Graphical abstract

Arithmetic mean plasma concentration versus time profiles for macitentan, aprocitentan and tadalafil during the first 24 hours and 216 hours after administration of Treatment A (FDC, fasted), Treatment B (FDC, fed), and Treatment C (free combination, fasted).

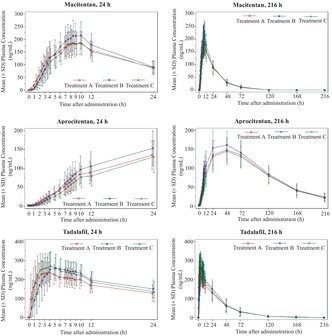

**Abbreviations**
AEs: adverse events
AUC: area under the plasma analyte concentration–time curve
*AUC*_∞_
: area under the plasma analyte concentration–time curve from time 0 to infinity
*AUC*_∞,ex_
: area under the plasma analyte concentration curve extrapolated from time 0 to infinity
*AUC*_last_
: area under the plasma analyte concentration–time curve from time 0 to time of the last quantifiable concentration
BMI: body mass index
CI: confidence interval
*C*_max_
: maximum observed plasma analyte concentration
CV: coefficient of variation
ECG: electrocardiogram
FDC: fixed‐dose combination
LS: least square
PAH: pulmonary arterial hypertension
PDE‐5: phosphodiesterase type 5
PK: pharmacokinetic(s)
QC: quality control
*r* ^2^_adj_
: coefficient of determination
SD: standard deviation
*t*_1/2_
: apparent terminal elimination half‐life
TEAE: treatment‐emergent adverse event
*t*_max_
: actual sampling time to reach the maximum observed plasma analyte concentration
*λ*_z_
: apparent terminal elimination rate constant

## 1 INTRODUCTION

Pulmonary arterial hypertension (PAH) is a chronic disease characterized by increased pulmonary vascular resistance, which, if not treated, results in right ventricular failure and death.^1^, ^2^

Macitentan is an orally active, nonpeptide, potent dual endothelin receptor antagonist with the brand name Opsumit® (macitentan 10 mg film‐coated tablet). It has been approved for the treatment of PAH at a dose of 10 mg once daily by health authorities including the United States Food and Drug Administration^3^ and the European Medicines Agency.^4^

Tadalafil is a selective phosphodiesterase type 5 (PDE‐5) inhibitor approved by health authorities including the United States Food and Drug Administration^5^ and the European Medicines Agency^6^ under the brand name Adcirca® (20 mg film‐coated tablet) for the treatment of PAH at a recommended dose of 40 mg for adult PAH patients.

The updated European Society of Cardiology/European Respiratory Society guidelines recommend that combination therapy of macitentan and tadalafil should be given as sequential or up‐front combination if treatment targets are not met.^7^ The Canadian Cardiovascular Society/Canadian Thoracic Society states in a Position Statement on Pulmonary Hypertension that a combination of PAH‐targeted medical therapies is standard of care for most PAH patients and strongly recommends initial dual oral combination therapy in intermediate‐risk treatment‐naïve patients.^8^

A single‐tablet formulation (fixed‐dose combination [FDC]) of macitentan/tadalafil provides PAH patients the efficacy and safety of a combined use of macitentan and a PDE‐5 inhibitor in one formulation facilitating a high level of compliance and a low risk of medication errors. An FDC of 10 mg macitentan and 40 mg tadalafil as single‐tablet combination therapy for PAH was already approved under the brand name Opsynvi® in Canada and Argentina for patients already receiving this treatment combination as separate tablets, based on bioequivalence studies.^9^, ^10^

However, it is recommended that patients who start initial combination therapy with macitentan and tadalafil, including those with mild to moderate hepatic impairment, should receive a starting dose of 20 mg tadalafil and then up‐titrate the dose to 40 mg tadalafil depending on tolerability and efficacy. The FDC of 10 mg macitentan and 20 mg tadalafil is intended for these patients.

Although tadalafil exposure (based on the AUC [area under the plasma analyte concentration–time curve]) in healthy participants increases proportionally over a dose range of 2.5–20 mg, a less than proportional increase in exposure of tadalafil is observed between 20 and 40 mg.^6^ Due to this non‐linearity in the pharmacokinetics (PK) of tadalafil, results of the bioequivalence and food effect studies conducted with the 40 mg dose of tadalafil cannot be extrapolated to the 20 mg tadalafil dose. Therefore, the rationale for this study was to demonstrate bioequivalence between the 10 mg macitentan/20 mg tadalafil FDC formulation and the co‐administered free combination of both drugs, and to evaluate the effect of fed (high‐fat, high‐calorie meal) versus fasted conditions on the systemic exposure of the FDC formulation in healthy adult participants.

The 10/20 mg FDC formulation used in this study was based on the qualitative composition of the 10/40 mg FDC.

## 2 METHODS

### 2.1 Study participants

Study participants were healthy adults aged ≥18 years and ≤55 years with a body mass index (BMI) between ≥18.5 kg m^−2^ and ≤30.0 kg m^−2^ and a body weight of no less than 50.0 kg. Key exclusion criteria included history of clinically significant loss of vision, known hereditary degenerative retinal disorders (including retinitis pigmentosa), history of priapism, conditions that predispose to priapism, anatomical deformation of the penis, and history of repeated fainting due to cardiac cause, collapse, syncope, orthostatic hypotension, or vasovagal reactions.

### 2.2 Study design

This was a single‐center (USA), randomized, open‐label, 3‐way crossover, single‐dose Phase 1 study in healthy adult participants. The primary and secondary objectives are shown in Table 1.

**TABLE 1:** Primary and secondary objectives.

Primary objectives	To demonstrate bioequivalence on the primary PK parameters between macitentan and tadalafil administered as an FDC of 10 mg macitentan/20 mg tadalafil and the co‐administered free combination of 10 mg macitentan (Opsumit®) and 20 mg tadalafil (Adcirca®) in fasted conditions in healthy adult participants
	2 To evaluate the effect of food on the primary PK parameters of macitentan and tadalafil administered as an FDC of 10 mg macitentan/20 mg tadalafil in fed versus fasted conditions in healthy adult participants
Secondary objectives	To investigate secondary PK parameters of macitentan and tadalafil, administered as an FDC in fasted and fed conditions or co‐administered as a free combination in fasted conditions in healthy adult participants
	2 To evaluate the safety and tolerability of macitentan and tadalafil administered as an FDC in fasted and fed conditions or co‐administered as a free combination in fasted conditions in healthy adult participants

Abbreviations: FDC, fixed‐dose formulation; PK, pharmacokinetic.

The study was conducted in accordance with the ethical principles that have their origin in the Declaration of Helsinki and that are consistent with Good Clinical Practices and applicable regulatory requirements. The protocol, protocol amendments, Informed Consent Form, Investigator's Brochure, and other relevant documents were reviewed and approved by the Institutional Review Board before the study was initiated. Written informed consent was obtained from each participant in the study prior to screening.

**FIGURE A1:**
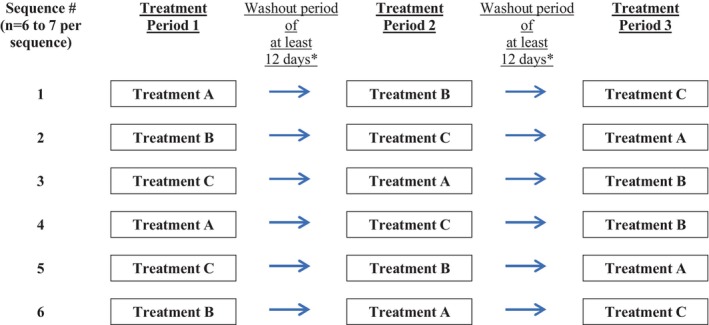
Schematic overview of the study.

Participants were randomly assigned to one of six treatment sequences according to a 6‐sequence, 3‐period design. In three sequential treatment periods, each participant received three treatments (Treatments A, B, and C), one in each period. A schematic overview of the study is available in the Figure A1.

Treatment A was a single oral film‐coated tablet of FDC with 10 mg macitentan/20 mg tadalafil administered under fasted conditions, Treatment B was a single oral film‐coated tablet of FDC with 10 mg macitentan/20 mg tadalafil administered under fed conditions (high‐fat, high‐calorie meal), and Treatment C was a free combination of a single oral film‐coated tablet of macitentan 10 mg (Opsumit®) and a single oral film‐coated tablet of tadalafil 20 mg (Adcirca®) administered under fasted conditions.

Participants received the study intervention only on Day 1 of each treatment period. Between the treatment periods, there were washout periods of at least 12 days. Participants were admitted to the study site in the morning of Day −1 of each treatment period and were discharged from the study site after the assessments of Day 4 (approximately 72 h after study intervention intake). Participants visited the study site on Days 6, 8, and 10 in each treatment period for assessments. End‐of‐period visits/end‐of‐study visits were performed approximately 216 h after the first study intervention intake of that period/after the last study intervention intake of the study.

The FDCs and the reference treatment of macitentan (Opsumit®) and tadalafil (Adcirca®) were provided by Actelion Pharmaceuticals.

### 2.3 Pharmacokinetic evaluations

#### 2.3.1 Sample collection and analytical methods

Blood samples (3 mL) were collected predose (within 2 h before study intervention intake) and at 0.5, 1, 1.5, 2, 2.5, 3, 3.5, 4, 5, 6, 7, 7.5, 8, 8.5, 9, 10, 12, 24, 48, 72, 120, 168, and 216 h postdose into the precooled appropriate dipotassium ethylenediaminetetraacetic acid‐containing collection tubes and kept on melting ice until processing. Within 30 min of collection, the tubes were centrifuged at approximately 1300 g for 10 min at 4°C. The plasma was then transferred into two labeled polypropylene tubes, one for the measurement of macitentan and its active metabolite aprocitentan (formerly designated as ACT‐132577), and one for the measurement of tadalafil. All samples were stored in an upright position below −20°C.

The plasma concentrations of macitentan, aprocitentan (combined assay), and tadalafil (separate assay) were measured at ICON Bioanalytical Laboratories, Assen, The Netherlands, using validated liquid chromatography methods with tandem mass spectrometry detection (Turbo‐ionspray in positive mode, 5500 series mass spectrometer for macitentan and aprocitentan and 5000 series for tadalafil [AB Sciex, CA, USA]) after protein precipitation with acetonitrile. Chromatographic separation was done with Agilent 1290 series liquid chromatography system. The column (Waters Acquity BEH C18 50 × 2.1 mm, 1.7 μm, Waters, Milford, USA) was kept at a temperature of 50°C. The mobile phase consisted of water (A) and acetonitrile (B). The gradient ran from ratio (A/B) 70/30 to 55/45 (% v/v) in 2.25 min. The flow rate was 1.0 mL/min. For tadalafil a Waters Acquity BEH C18 30 × 2.1 mm, 1.7 μm column was used, at a temperature of 55°C. The mobile phase consisted of 0.1% formic acid in water (C) and acetonitrile (D). The gradient ran from ratio (C/D) 70/30 to 65/35 (% v/v) in 0.5 min, at a flowrate of 1.5 mL/min. Stable isotope labeled analogues of the compounds were used as internal standards (^2^H_4_ for macitentan and aprocitentan and ^13^C^2^H_3_ for tadalafil). For the tandem mass spectrometry detection, the mass transitions (m/z) of macitentan and aprocitentan were 589.0 → 201.0 and 547.0 → 201.0 and for the respective internal standards 595.0 → 207.0 and 553.0 → 207.0. For tadalafil this was 390.1 → 268.1 and for the internal standard 394.2 → 272.1. The internal standards were used to calculate peak area response ratios that were used for quantification (linear regression using weighting factor 1/*x* ^2^). The assay range for macitentan and aprocitentan was 1.00–2000 ng mL^−1^. The range for tadalafil was 0.500–1000 ng mL^−1^. The results from calibration samples and quality control (QC) samples demonstrated acceptable performance of the method throughout the experimental period. Data on performance of the method and stability indicate that the plasma concentration results of macitentan, aprocitentan, and tadalafil are reliable.

Stability of macitentan and aprocitentan, in the presence of tadalafil, in plasma samples was demonstrated for up to 26 h at room temperature, 371 days at −20°C, and for five freeze/thaw cycles.

Stability of tadalafil, in the presence of macitentan and aprocitentan, in plasma samples was demonstrated for up to 24 h at room temperature, 259 days at −20°C, and for five freeze/thaw cycles.

The descriptive statistics of the QC samples showed that the interbatch precision was ≤10.7% for macitentan, ≤12.1% for aprocitentan, and ≤5.4% for tadalafil, whereas the interbatch bias was within the range of −3.2% to 4.7%, −4.0% to 3.7%, and −3.5% to 2.2% for these analytes, respectively.

Incurred sample analysis was performed for both methods. For macitentan and aprocitentan, 6.9% of a total of 2781 samples were reanalyzed, and 97.9% and 100% were within the acceptance criteria for macitentan and aprocitentan, respectively (relative difference from the original result should be less than or equal to ±20%). For tadalafil, 6.8% of a total of 2780 samples were reanalyzed, and 98.9% were within the acceptance criteria.

#### 2.3.2 Pharmacokinetic analyses

All participants who received at least one dose of any study intervention and had at least one sample with evaluable plasma concentrations were included in the PK analyses and in the descriptive statistics.

Calculation of the apparent terminal elimination half‐life (*t*_1/2_), the apparent terminal elimination rate constant (*λ*_z_), and the area under the plasma analyte concentration–time curve from time 0 to infinity (*AUC*_∞_) required, at least, three data points (not including the maximum observed plasma analyte concentration [*C*_max_]) and a coefficient of determination (*r* ^2^_adj_) of 0.900. In cases where *r* ^2^_adj_ was <0.900, *t*_1/2_, *λ*_z_, and *AUC*_∞_ were reported but excluded from the descriptive analysis and statistical evaluation. The same exclusion criteria were applied for *AUC*_∞_ and related PK parameters when the area under the concentration curve extrapolated from time 0 to infinity (*AUC*_∞,ex_) was >20%. For sampling times that deviated by >20% from the scheduled (nominal) time, the plasma concentration was excluded from descriptive statistics but included in the estimation of PK parameters using the actual sampling time.

The PK data from the different treatment sequences were grouped with sequence, period, and treatment identification (Treatment A, B, and C). For each treatment, descriptive statistics were calculated for plasma concentrations of macitentan, aprocitentan, and tadalafil at each applicable time point specified and for the derived plasma PK parameters. Statistics included the sample size (*n*), mean, standard deviation (SD), coefficient of variation (CV), geometric mean, median, minimum, and maximum.

The analyses were performed on log‐transformed PK parameters of interest. The primary PK parameters of interest for both the bioequivalence and food effect testing were *C*_max_, area under the plasma analyte concentration–time curve from time 0 to time of the last quantifiable concentration (*AUC*_last_), and *AUC*_∞_ of macitentan, aprocitentan, and tadalafil. The least square (LS) means of the log‐transformed primary PK parameters for each treatment were estimated with a linear mixed‐effects model, with treatment, sequence, and period as fixed effects, and participant as a random effect. Bioequivalence was assessed by the difference between the LS means of Treatment A (FDC [test]) and Treatment C (free combination [reference]) and the food effect was assessed by the difference between the LS means of Treatment B (fed [test]) and Treatment A (fasted [reference]). A 90% confidence interval (CI) was constructed for the difference between the LS means.

Both the difference between the LS means and the 90% CIs were retransformed to the original scale to obtain an estimated ratio of geometric means and corresponding 90% CIs.

For acceptance of bioequivalence to individual agents given as separate tablets, the entire CI limits for the ratio of geometric means for Treatments A versus C had to fall within 80.00% and 125.00%.^11^ Absence of food effect was concluded if the entire CI limits for the ratio of means for Treatments B versus A fell within 80.00% and 125.00%. An intersection–union test was conducted to address bioequivalence and absence of food effect using the above‐mentioned 90% CI within the 80.00% to 125.00% range. Intersection–union testing does not require multiplicity testing.

#### 2.3.3 Safety evaluation

All participants who received at least one dose of any of the study interventions were included in the safety and tolerability analysis. The baseline for all laboratory evaluations, vital signs, and electrocardiogram (ECG) measurements were defined as the last observation prior to the start of the first study intervention administration in the first treatment period.

The safety data from the different treatment sequences were grouped with sequence, period, and treatment identification (Treatment A, B, and C).

#### 2.3.4 Sample size calculation

Using an estimated intraparticipant CV of 15% for the primary PK parameters of tadalafil and macitentan, a sample size of 30 completed participants was expected to be sufficient to conclude bioequivalence or food effect with 90% power, when the true ratio of treatment equaled 90% or 110%. The intraparticipant CV of 15% was based on results from a pilot relative bioavailability study (unpublished data on file, NCT04540744, Actelion Pharmaceuticals Ltd, a Johnson & Johnson Company, Allschwil, Switzerland). A total of 40 participants were planned to be enrolled in the study to ensure that about 30 participants completed all assigned treatments. If participants discontinued the study prior to study intervention administration in the first treatment period, additional participants could be recruited to aim for 40 participants starting treatment. The first 10 participants to discontinue were not to be replaced. If additional participants discontinued for reasons other than adverse events (AEs), they could be replaced. Additional participants would start in treatment period 1 and receive treatments according to the same sequence as the discontinued participant who was replaced.

#### 2.3.5 Nomenclature of targets and ligands

Key protein targets and ligands in this article are hyperlinked to corresponding entries in http://www.guidetopharmacology.org, the common portal for data from the IUPHAR/BPS Guide to PHARMACOLOGY,^12^ and are permanently archived in the Concise Guide to PHARMACOLOGY 2023/24.^13^

## 3 RESULTS

### 3.1 Study participants

A total of 40 participants were enrolled and randomly assigned to one of six treatment sequences. All 40 (100.0%) participants were included in the safety analysis. All 40 (100.0%) participants received at least one dose of the study drugs and had at least one plasma concentration data value after study drug intake and were therefore included in the descriptive statistical analysis on plasma concentrations and PK parameters (PK data analysis set). A total of 37 (92.5%) participants received all planned doses of the study drugs and were therefore included in the inferential statistical PK analysis (PK data statistical analysis set). Of the 40 participants enrolled, 37 (92.5%) participants completed the study. Three (7.5%) participants withdrew prematurely from the study. One participant (planned treatment sequence B‐C‐A) was withdrawn from the study due to a treatment‐emergent adverse event (TEAE) after having completed Treatments B and C. One participant (planned treatment sequence A‐B‐C) was withdrawn from the study due to a TEAE after having completed Treatment A. One participant (planned treatment sequence B‐C‐A) was withdrawn from the study due to a positive urine drug test after having completed Treatments B and C.

### 3.2 Demographics and baseline characteristics

Overall, 16 (40.0%) participants were women, and 24 (60.0%) participants were men (Table 2). The mean (SD) age was 30.9 (8.86) years, and the mean (SD) BMI was 24.51 (3.029) kg m^−2^.

**TABLE 2:** Demographic and baseline characteristics, safety analysis set.

Treatment sequence	A‐B‐C	B‐A‐C	C‐A‐B	A‐C‐B	B‐C‐A	C‐B‐A	Total
*n*	7	6	7	7	6	7	40
Sex							
Women, *n* (%)	2 (28.6%)	4 (66.7%)	2 (28.6%)	2 (28.6%)	3 (50.0%)	3 (42.9%)	16 (40.0%)
Men, *n* (%)	5 (71.4%)	2 (33.3%)	5 (71.4%)	5 (71.4%)	3 (50.0%)	4 (57.1%)	24 (60.0%)
Arithmetic mean (SD); min‐max							
Age, years	29.0 (5.10); 21–36	28.3 (8.14); 18–38	32.9 (10.11); 22–47	30.9 (8.19); 22–46	29.8 (13.61); 19–53	34.0 (8.83); 21–44	30.9 (8.86); 18–53
Weight at the baseline, kg	68.40 (8.933); 59.6–82.9	65.13 (8.424); 52.5–76.3	72.97 (13.456); 51.1–90.2	72.80 (18.016); 50.5–105.2	73.83 (16.528); 52.5–91.9	76.37 (12.485); 61.9–92.5	71.69 (13.110); 50.5–105.2
Height at the baseline, cm	166.4 (7.63); 156–179	168.3 (12.01); 153–184	171.3 (10.92); 153–182	174.6 (9.57); 163–188	171.8 (13.85); 155–186	171.0 (10.23); 153–180	170.6 (10.38); 153–188
BMI at the baseline, kg m^−2^	24.69 (2.465); 21.0–28.3	23.05 (2.957); 19.8–28.1	24.77 (3.445); 21.0–29.5	23.60 (3.674); 18.5–29.8	24.72 (2.499); 21.9–28.1	26.07 (3.099); 20.1–28.8	24.51 (3.029); 18.5–29.8

*Note*: Treatment A: single oral film‐coated tablet of FDC of macitentan/tadalafil (10/20 mg) in fasted conditions. Treatment B: single oral film‐coated tablet of FDC of macitentan/tadalafil (10/20 mg) in fed conditions (high‐fat/high‐calorie breakfast). Treatment C: single oral film‐coated tablet of macitentan 10 mg (Opsumit®) and single oral film‐coated tablet of tadalafil 20 mg (Adcirca®) in fasted conditions.

Abbreviations: BMI, body mass index; FDC, fixed‐dose combination; max, maximum; min, minimum; SD, standard deviation.

### 3.3 Pharmacokinetics

#### 3.3.1 Macitentan and aprocitentan

**FIGURE 1:**
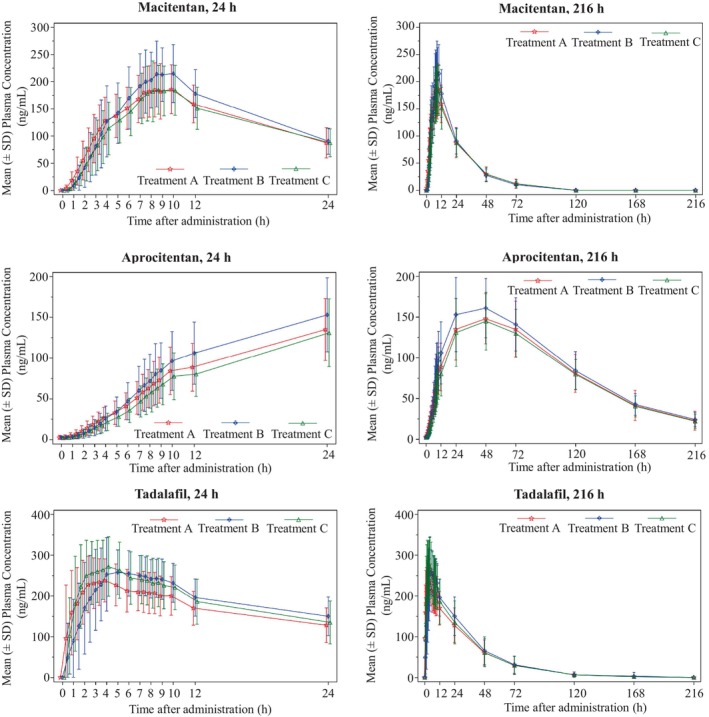
Arithmetic Mean Plasma Concentration Versus Time Profiles for Macitentan, Aprocitentan and Tadalafil During the First 24 h and 216 h After the administration of Treatment A (FDC, Fasted), Treatment B (FDC, Fed), and Treatment C (Free combination, Fasted), Pharmacokinetics Data Analysis Set. FDC, fixed‐dose combination; *n*, number of participants; SD, standard deviation. Data are plotted on a linear scale. *n* for Macitentan: 38 (Treatment A) and 39 (Treatments B and C); *n* for Aprocitentan; 35 (Treatment A) and 38 (Treatments B and C); *n* for Tadalafil: 38 (Treatments A and C) and 39 (Treatment B). Treatment A: single oral film‐coated tablet of FDC of 10 mg macitentan/20 mg tadalafil in fasted conditions. Treatment B: single oral film‐coated tablet of FDC of 10 mg macitentan/20 mg tadalafil in fed conditions (high‐fat/high‐calorie breakfast). Treatment C: single oral film‐coated tablet of macitentan 10 mg (Opsumit®) and single oral film‐coated table of tadalafil 20 mg (Adcirca®) in fasted stated.

For both macitentan and aprocitentan, *C*_max_ and AUCs were similar following the administration of Treatment A (FDC, fasted), Treatment B (FDC, fed), and Treatment C (free combination, fasted; Table 3; Figure 1).

**TABLE 3:** Summary of pharmacokinetic results of macitentan, aprocitentan, and tadalafil by treatment, pharmacokinetics data analysis set.

Geometric means (SD); *t*_max_: median (min‐max)	Macitentan			Aprocitentan			Tadalafil		
	Treatment A (FDC, fasted)	Treatment B (FDC, fed)	Treatment C (free comb., fasted)	Treatment A (FDC, fasted)	Treatment B (FDC, fed)	Treatment C (free comb., fasted)	Treatment A (FDC, fasted)	Treatment B (FDC, fed)	Treatment C (free comb., fasted)
*n*	38^a^	39	39^b^	35	38	35^c^	38^d^	39^e^	38^f^
*C*_max_ (ng mL^−1^)	203 (45.1)	238 (60.3)	197 (53.3)	153 (37.6)	169 (39.1)	150 (36.7)	289 (85.1)	314 (75.0)	312 (72.8)
*AUC*_72h_ (ng h mL^−1^)	4942 (1121)	5152 (1159)	4792 (1199)	8662 (1976)	9543 (2335)	8332 (2160)	7532 (2137)	8456 (2200)	7923 (2555)
*AUC*_last_ (ng h mL^−1^)	5210 (1384)	5361 (1344)	5044 (1409)	18 784 (4607)	19 983 (4417)	18 104 (4046)	8709 (3149)	9824 (3248)	9154 (3649)
*AUC*_∞_ (ng h mL^−1^)	5313 (1388)	5454 (1339)	5155 (1410)	20 623 (5802)	21 976 (4992)	19 966 (4765)	8857 (3139)	9644 (3259)	9109 (3530)
*t*_max_ (h)	8.50 (3.50–24.00)	9.00 (3.50–12.02)	8.50 (7.00–12.00)	48.00 (24.00–72.00)	48.00 (24.00–72.00)	48.00 (24.00–72.00)	3.00 (0.50–12.00)	5.00 (1.50–10.02)	2.81 (0.50–10.07)
*t*_1/2_ (h)	16.0 (3.4)	15.2 (3.8)	16.4 (3.6)	52.2 (8.8)	54.4 (10.4)	52.4 (10.2)	21.5 (6.9)	20.1 (6.3)	20.6 (6.3)
*C*_last_ (ng mL^−1^)	4.62 (3.13)	4.42 (3.12)	4.86 (2.73)	22.8 (11.8)	24.2 (8.93)	22.1 (9.04)	1.92 (1.69)	2.76 (7.11)	1.71 (1.46)

*Note*: Treatment A: single oral film‐coated tablet of FDC of 10 mg macitentan/20 mg tadalafil in fasted conditions. Treatment B: single oral film‐coated tablet of FDC of 10 mg macitentan/20 mg tadalafil in fed conditions (high‐fat, high‐calorie breakfast). Treatment C: single oral film‐coated tablet 10 mg macitentan (Opsumit®) and single oral film‐coated tablet of tadalafil 20 mg (Adcirca®) in fasted conditions.

Abbreviations: *AUC*_72h_, area under the plasma analyte concentration–time curve from time 0 to 72 h postdose; *AUC*_∞_, area under the plasma analyte concentration–time curve from time 0 to infinity; *AUC*_last_, area under the plasma analyte concentration–time curve from time 0 to time of the last quantifiable concentration; *C*_last_, last observed measurable plasma analyte concentration; *C*_max_, maximum observed plasma analyte concentration; comb., combination; FDC, fixed‐dose combination; max, maximum; min, minimum; SD, standard deviation; *t*_1/2_, apparent terminal elimination half‐life; *t*_max_, actual sampling time to reach the maximum observed plasma analyte concentration.

^a^ *n* = 37 for *AUC*_∞_ and *t*_1/2_.

^b^ *n* = 38 for *AUC*_72h_, *AUC*_last_, *AUC*_∞_, *t*_1/2_, and *C*_last_.

^c^ *n* = 33 for *AUC*_∞_; *n* = 34 for *t*_1/2_.

^d^ *n* = 37 for *AUC*_∞_, *t*_1/2_, and *C*_last_.

^e^ *n* = 38 for *AUC*_72h_, *AUC*_∞_, *t*_1/2_, and *C*_last_.

^f^ *n* = 37 for *AUC*_72h_, *AUC*_∞_, *t*_1/2_, and *C*_last_.

When comparing Treatment A (FDC, fasted) and Treatment C (free combination, fasted), the 90% CIs for the geometric mean ratios of *C*_max_, *AUC*_last_, and *AUC*_∞_ for macitentan and aprocitentan were within the bioequivalence limits (80% to 125%; Table 4). Therefore, the FDC formulation can be considered bioequivalent to the free combination for macitentan and aprocitentan.

**TABLE 4:** Results of bioequivalence and food effect determination, pharmacokinetics data statistical analysis set.

	% ratio of geometric means (90% CI)					
	Bioequivalence: Treatment A versus C			Food effect: Treatment B versus A		
	Macitentan (*N* = 37^a^)	Aprocitentan (*N* = 32^b^)	Tadalafil (*N* = 36^c^)	Macitentan (*N* = 37^a^)	Aprocitentan (*N* = 32^b^)	Tadalafil (*N* = 36^c^)
*C*_max_ (ng mL^−1^)	103.34 (97.19, 109.88)	99.75 (96.23, 103.39)	91.86 (85.02, 99.24)	116.13 (109.20, 123.49)	112.78 (108.79, 116.93)	110.79 (102.54, 119.71)
*AUC*_last_, (ng h mL^−1^)	103.97 (100.34, 107.74)	101.66 (98.59, 104.81)	96.13 (90.24, 102.40)	100.45 (96.94, 104.10)	106.53 (103.30, 109.86)	109.11 (102.38, 116.28)
*AUC*_∞_, (ng h mL^−1^)	104.18 (100.68, 107.80)	101.32 (98.15, 104.59)	96.06 (90.97, 101.43)	100.29 (96.91, 103.79)	107.08 (103.70, 110.56)	110.21 (104.37, 116.39)

*Note*: Treatment A: single oral film‐coated tablet of FDC of 10 mg macitentan/20 mg tadalafil in fasted conditions. Treatment B: single oral film‐coated tablet of FDC of 10 mg macitentan/20 mg tadalafil in fed conditions (high‐fat, high‐calorie breakfast). Treatment C: single oral film‐coated tablet 10 mg macitentan (Opsumit®) and single oral film‐coated tablet of tadalafil 20 mg (Adcirca®) in fasted conditions.

Abbreviations: *AUC*_∞_, area under the plasma analyte concentration–time curve from time 0 to infinity; *AUC*_last_, area under the plasma analyte concentration–time curve from time 0 to time of the last quantifiable concentration; CI, confidence interval; *C*_max_, maximum observed plasma analyte concentration; FDC, fixed‐dose combination.

^a^ *n* = 36 for *AUC*_∞_.

^b^ *n* = 31 for *AUC*_∞_.

^c^ *n* = 30 for *AUC*_last_.

Administering the FDC with a high‐fat, high‐calorie meal (Treatment B) resulted in negligible increase in exposure (based on *AUC*_last_, *AUC*_∞_, and *C*_max_) for macitentan and aprocitentan when compared with the fasted state (Treatment A; Table 4).

#### 3.3.2 Tadalafil

For tadalafil, *C*_max_ and *AUC*_s_ were similar following the administration of Treatment A (FDC, fasted), Treatment B (FDC, fed), and Treatment C (free combination, fasted; Table 3, Figure 1).

When comparing Treatments A (FDC, fasted) and C (free combination, fasted), the 90% CIs for the geometric mean ratios of *C*_max_, *AUC*_last_, and *AUC*_∞_ for tadalafil were within the bioequivalence limits (80% to 125%; Table 4). Therefore, the FDC formulation can be considered bioequivalent to the free combination for tadalafil.

Administering the FDC with a high‐fat, high‐calorie meal (Treatment B) resulted in negligible increase in exposure (based on *AUC*_last_, *AUC*_∞_, and *C*_max_) for tadalafil when compared with the fasted state (Treatment A; Table 4). The absorption rate was also slightly delayed with a median actual sampling time to reach the maximum observed plasma analyte concentration (*t*_max_) of 5.00 h for Treatment B versus 3.00 h for Treatment A and 2.81 h for Treatment C (Table 3).

### 3.4 Safety

The administration of 10/20 mg macitentan/tadalafil was generally safe and well tolerated in healthy adult participants, both when administered under either fasted or fed conditions as a single oral dose of an FDC formulation (Treatments A and B, respectively) or when administered under fasted conditions as a free combination of a single oral dose of 10 mg macitentan (Opsumit®) and a single oral dose of 20 mg tadalafil (Adcirca®; Treatment C).

No serious TEAEs or deaths were reported during the study. Two (5.0%) participants terminated the study prematurely due to a TEAE: one participant under Treatment C (free combination, fasted) due to influenza and one participant under Treatment A (FDC, fasted) due to blood creatine phosphokinase increased after strenuous physical activity. Neither event was considered related to the study intervention. Overall, 27 (67.5%) participants experienced at least one AE during the screening and treatment phases. The incidence of TEAEs was 10 (26.3%) participants following the administration of Treatment A (FDC, fasted), 15 (38.5%) participants following the administration of Treatment B (FDC, fed), and 20 (51.3%) participants following the administration of Treatment C (free combination, fasted).

The most frequently reported TEAE by preferred term was headache (13 [32.5%] participants; Table 5). Other frequently reported TEAEs (in at least 3 participants) included back pain, myalgia, nausea, pain in extremity, dizziness, and vomiting.

**TABLE 5:** Treatment‐emergent adverse events by preferred term occurring in >1 participant; safety analysis set.

	Treatment A (FDC, fasted)	Treatment B (FDC, fed)	Treatment C (free comb., fasted)	Total
*n*	38	39	39	40
Preferred term, *n* (%)				
Headache	3 (7.9%)	6 (15.4%)	6 (15.4%)	13 (32.5%)
Back pain	2 (5.3%)	3 (7.7%)	7 (17.9%)	11 (27.5%)
Myalgia	2 (5.3%)	3 (7.7%)	4 (10.3%)	7 (17.5%)
Nausea	2 (5.3%)	2 (5.1%)	2 (5.1%)	6 (15.0%)
Pain in extremity	1 (2.6%)	0	4 (10.3%)	4 (10.0%)
Dizziness	2 (5.3%)	0	1 (2.6%)	3 (7.5%)
Vomiting	1 (2.6%)	1 (2.6%)	1 (2.6%)	3 (7.5%)
Blood creatine phosphokinase increased	1 (2.6%)	1 (2.6%)	0	2 (5.0%)
Influenza	0	0	2 (5.1%)	2 (5.0%)

*Note*: Participants are counted only once for any given event, regardless of the number of times they actually experienced the event. Adverse events are coded using MedDRA Version 24.1. The denominator for percentage calculations is the number of participants in the safety analysis set for that treatment. Sorting order: Descending by the number of participants by preferred term in the Total column. Treatment A: single oral film‐coated tablet of FDC of 10 mg macitentan/20 mg tadalafil in fasted conditions. Treatment B: single oral film‐coated tablet of FDC of 10 mg macitentan/20 mg tadalafil in fed conditions (high‐fat, high‐calorie breakfast). Treatment C: single oral film‐coated tablet of macitentan 10 mg (Opsumit®) and single oral film‐coated tablet of tadalafil 20 mg (Adcirca®) in fasted conditions.

Abbreviations: comb., combination; FDC, fixed‐dose combination.

The majority of TEAEs were mild or moderate in severity. No participants experienced severe TEAEs.

Overall, the mean changes in clinical laboratory values, vital signs, and ECG measurements were similar across the treatment groups and generally not considered clinically significant by the investigator.

## 4 DISCUSSION

This Phase 1 study in healthy adult participants showed that the FDC of 10 mg macitentan/20 mg tadalafil in a single tablet was bioequivalent to the free combination of Opsumit® (macitentan 10 mg film‐coated tablet) and Adcirca® (tadalafil 20 mg film‐coated tablet). When comparing Treatments A (FDC, fasted) and C (free combination, fasted), the 90% CIs for the geometric mean ratios of *C*_max_, *AUC*_last_, and *AUC*_∞_ were within the bioequivalence limits. Similar results were obtained in previous studies for the 10/40 mg FDC formulation.^9^, ^10^ When administered in the free combination, the PK profiles of macitentan and tadalafil were also similar to those observed in previous studies.^14^, ^15^, ^16^

In addition, this study showed that there was no food effect on plasma exposure with the FDC 10/20 mg formulation. The increase in exposure after administering the FDC with a high fat, high calorie meal was negligible, and the small delay in the rate of absorption was similar to that observed with the 10/40 FDC formulation^10^ and not considered clinically relevant.

The administration of 10 mg macitentan/20 mg tadalafil was generally safe and well tolerated in healthy adult participants, both when administered as a single oral dose of an FDC formulation under fasted or fed conditions, or as the free combination of both single oral doses under fasted conditions.

## 5 CONCLUSIONS

This study demonstrated bioequivalence between the FDC formulation of 10 mg macitentan/20 mg tadalafil as a single tablet and the free combination of both drugs as separate tablets in healthy participants, suggesting that the 10/20 mg FDC formulation might be an alternative to the free combination for patients who start initial combination therapy and patients who cannot up‐titrate to 40 mg tadalafil. A 10/20 mg FDC would reduce the pill burden for these patients. The absence of a food effect indicates that the 10/20 mg FDC formulation, like the 10/40 mg FDC formulation, can be taken with or without food, like the individual components. The administration of 10/20 mg of macitentan/tadalafil was generally safe and well tolerated in healthy adult participants. There were no apparent differences in the safety profile between the FDC formulation and the free combination.

## AUTHOR CONTRIBUTIONS

AS was responsible for the study implementation and had direct responsibility for the subjects. JF was responsible for the PK analysis and for the interpretation of the data. HS was responsible for the bioanalytical activities. JN was responsible for the statistical analysis. NG, DLC, and DC were responsible for the interpretation of the data. All authors reviewed, contributed to, and approved the final version of the manuscript.

## FUNDING INFORMATION

Janssen Research & Development.

## CONFLICT OF INTEREST STATEMENT

JLF, NG, JN, and DLC are employed by Johnson & Johnson and own sponsor stocks/stock options. HS is employed by Johnson & Johnson. DC is employed by Actelion Pharmaceuticals Ltd., a Janssen Pharmaceutical Company of Johnson & Johnson, and owns sponsor stocks/stock options. AS is employed by ICON.

## ETHICS STATEMENT

The study was conducted in accordance with the ethical principles that have their origin in the Declaration of Helsinki and that are consistent with Good Clinical Practices and applicable regulatory requirements. The protocol, protocol amendments, Informed Consent Form, Investigator's Brochure, and other relevant documents were reviewed and approved by the Institutional Review Board before the study was initiated.

## PRINCIPAL INVESTIGATOR STATEMENT

The authors confirm that the Principal Investigator for this paper is Ahad Sabet, and that he had direct clinical responsibility for patients.

## PATIENT CONSENT STATEMENT

Written informed consent was obtained from each participant in the study prior to screening.

## PERMISSION TO REPRODUCE MATERIAL FROM OTHER SOURCES

Not applicable.

## CLINICAL TRIAL REGISTRATION

ClinicalTrials.gov Identifier: NCT05236231.

## Data Availability

The data sharing policy of the Sponsor is available at: https://www.janssen.com/clinical‐trials/transparency. As noted on this site, requests for access to the study data can be submitted through Yale Open Data Access (YODA) Project site at http://yoda.yale.edu.
